# Ruminal metagenomic libraries as a source of relevant hemicellulolytic enzymes for biofuel production

**DOI:** 10.1111/1751-7915.13269

**Published:** 2018-04-17

**Authors:** Estrella Duque, Abdelali Daddaoua, Baldo F. Cordero, Zulema Udaondo, Carlos Molina‐Santiago, Amalia Roca, Jennifer Solano, Eduarda Molina‐Alcaide, Ana Segura, Juan‐Luis Ramos

**Affiliations:** ^1^ Estación Experimental del Zaidín (CSIC) Calle Profesor Albareda, 1 18008 Granada Spain; ^2^ Department of Biotechnology Abengoa Research Campus Palmas Altas, Avenida de la Energia 1 41014 Seville Spain; ^3^ Faculty of Pharmacy Department of Pharmacology University of Granada 18071 Granada Spain; ^4^ BioIliberis R&D Polígono Juncaril Calle Capileira 7 18220 Albolote Granada Spain

## Abstract

The success of second‐generation (2G) ethanol technology relies on the efficient transformation of hemicellulose into monosaccharides and, particularly, on the full conversion of xylans into xylose for over 18% of fermentable sugars. We sought new hemicellulases using ruminal liquid, after enrichment of microbes with industrial lignocellulosic substrates and preparation of metagenomic libraries. Among 150 000 fosmid clones tested, we identified 22 clones with endoxylanase activity and 125 with β‐xylosidase activity. These positive clones were sequenced *en masse,* and the analysis revealed open reading frames with a low degree of similarity with known glycosyl hydrolases families. Among them, we searched for enzymes that were thermostable (activity at > 50°C) and that operate at high rate at pH around 5. Upon a wide series of assays, the clones exhibiting the highest endoxylanase and β‐xylosidase activities were identified. The fosmids were sequenced, and the corresponding genes cloned, expressed and proteins purified. We found that the activity of the most active β‐xylosidase was at least 10‐fold higher than that in commercial enzymatic fungal cocktails. Endoxylanase activity was in the range of fungal enzymes. Fungal enzymatic cocktails supplemented with the bacterial hemicellulases exhibited enhanced release of sugars from pretreated sugar cane straw, a relevant agricultural residue.

## Introduction

Rising energy consumption, depletion of fossil fuels and increased environmental concern have placed the focus of energy generation on the production of liquid biofuels from agricultural residues and municipal solid wastes, and in particular, large efforts have been devoted to the production of bioethanol, this ethanol is known as 2G ethanol in contrast with ethanol produced from grain or sugarcane known as 1G (Dashtban *et al*., 2009; Ramos *et al*., 2016; Valdivia *et al*., 2016). Ethanol production from agricultural residues (i.e. corn stover, bagasse, sugar cane straw, woody biomass) is a sustainable and environmental friendly alternative to fossil fuels (Balan, 2014; Han *et al*., 2015; Quinn and Davis, 2015). However, one of the major obstacles to the industrial‐scale production of fuels from lignocellulosic materials is the inefficient breakdown of plant material imparted by their recalcitrant nature (Hermsworth *et al*., 2015; Blombe *et al*., 2017). Efficient hydrolysis of lignocellulosic material requires intensive pretreatments (physical, chemical or a combination of both) to make cellulose and hemicellulose available for enzymatic hydrolysis (Bothast and Saha, 1997; Álvarez *et al*., 2016; Valdivia *et al*., 2016). Degradation of pretreated lignocellulosic residues requires the synergistic action of several enzymes to breakdown cellulose and hemicellulose. Three types of enzymes endo‐β‐1,4‐glucanases, *exo*‐β‐1,4‐glucanases (also known as cellobiohydrolases) and β‐1,4‐glucosidases are necessary for the efficient breakdown of cellulose to glucose (Rubin, 2008). The degradation of hemicellulose is enzymologically more complex and requires the concerted action of backbone‐depolymerizing enzymes (endoxylanases and β‐xylosidases), as well as accessory enzymes to hydrolyse the side‐chains on the xylan backbone (α‐l‐arabinofuranosidases, acetyl xylan esterases, feruloyl esterases and α‐glucuronidases) (Oguntimein and Moo‐Young, 1991; Herrmann *et al*., 1997; Belfaquih and Penninckx, 2000; Kiss and Kiss, 2000; Polizeli *et al*., 2005; Schagerlof *et al*., 2007; Harris and De Bolt, 2010; Borin *et al*., 2015; Álvarez *et al*., 2016). The two most abundant sugars resulting from cellulose and hemicellulose hydrolysis are always glucose and xylose, although the proportion varies with the source of the biomass, that is 70–90% of total sugars is glucose while 10–20% is xylose. In the 2G bioethanol industry, the achievement of target ethanol yields [i.e. 70–75 ethanol gallons/dry US short ton (DUSST)] requires both efficient production of monosaccharides from cellulose and hemicellulose and C5 yeasts that simultaneously ferment xylose and glucose (Dos Santos *et al*., 2016; Caballero and Ramos, 2017; Nakanishi *et al*., 2017). While fungal cellulases efficiently decompose, cellulose and industrial cocktails perform optimally with up to 75–80% of cellulose being transformed into glucose, the breakdown of hemicellulose is compromised by the limited amount of endoxylanases and β‐xylosidases in the cocktails. The high cost of these hemicellulolytic enzymes is one of the major bottlenecks for the production of 2G ethanol (Gao *et al*., 2011; Valdivia *et al*., 2016). To improve the performance of these enzymes, site‐directed mutagenesis and chemical mutagenesis have been used to generate lignocellulose‐degrading variant enzymes with enhance performance (Wen *et al*., 2009; Mohanram *et al*., 2013); however, the limited success of these approaches led to new trends that aim to retrieve enzymes from naturally evolved biomass‐degrading microbial communities, what offers great promises for the identification of new enzymes (Rubin, 2008; Hess *et al*., 2011; Chistoserdova, 2014; Ferrer *et al*., 2016; Alcalde, 2017).

The microbial world is considered the largest reservoir of genes encoding enzymatic catalysts; however, this wealth of catalytic potential remains largely unexplored because more than 99% of the microbes from soils, freshwaters, oceans and other niches cannot be grown in the laboratory (Pham and Kim, 2012; Stewart, 2012; Coughlan *et al*., 2015). Advances in cultivation‐independent DNA recovery methods now allow scientists to explore any niche on Earth and to extract the ‘hidden’ genetic diversity through large‐scale DNA sequencing (Rees *et al*., 2003; Handelsman, 2004; Kowalchuk *et al*., 2007; Rashid and Stingl, 2015). In fact, the bioprospecting analysis from several environments, including algal (Han *et al*., 2015) and crustacean seawaters (Ekborg *et al*., 2005), termite guts (Warnecke *et al*., 2007), poplar wood chips and hardwood forest decomposed by fungi and bacteria, have led to the identification of new genes and organisms with improved cellulolytic activities (Nguyen *et al*., 2012; Van der Lelie *et al*., 2012; Álvarez *et al*., 2016). One of the best‐characterized examples of an effective complex biomass‐degrading community is that harboured within the rumen of a number of herbivores (Nguyen *et al*., 2012). The microbiota inhabiting the rumen is characterized by a high cell density and wide diversity that includes anaerobic bacteria, fungi, protozoa and archaea, all of which are capable of secreting enzymes that degrade plant cell walls and thus represent an important source for hydrolytic enzymes (Walton, 1994; Tajima *et al*., 2000; Kamra, 2005; Gong *et al*., 2012; Nguyen *et al*., 2012; Gruninger *et al*., 2014; McCann *et al*., 2014).

Lignocellulosic‐degrading enzymes are not only relevant for biofuel production but they have also gained use in several industries, including biopulping of wood, treatment of animal feeds to increase digestibility, juice processing for improved clarification, flour handling modification for baking and as fibre softeners in textile preparation (Rashamuse *et al*., 2013). Therefore, the interest in cellulases/hemicellulases is wider than the sector of bioenergy, and in particular, the use of these enzymes by the detergent industry can be as relevant as that of 2G ethanol at present.

The main purpose of this study was to unveil the catalytic potential of rumen microbes to breakdown hemicellulose. To this end, three Murciano–Granadina goats were used as a source of ruminal liquid which was then supplemented with relevant 2G technology substrates [pretreated corn stover (PCS) and pretreated sugar cane straw (PSCS), and an abundant agricultural residue in Mediterranean countries: olive branches and leaves (OL)]. The enriched microbial milieu was used as a source of DNA to construct metagenomic libraries. We identified highly active novel bacterial hemicellulases using a combination of activity‐based screening and DNA sequencing. We then showed that these bacterial enzymes are active on industrial substrates and are potentially useful to replace the same enzymes in current 2G fungal cocktails. This kind of biotechnological approach aims to contribute to achievement of sustainability goals (Timmis *et al*., 2017).

## Results and discussion

### Construction and functional screening of ruminal metagenomic libraries

Ruminal liquid was harvested as described in Appendix S1 and used for *in vitro* microbial enrichment with PCS, PSCS and OL as a source of carbon and energy. The hemicellulose content of PCS was 3.5 g 100 g^−1^ dry matter, whereas for PSCS and OL, the hemicellulose content was around 15 g 100 g^−1^ dry matter (see Table S1). After 72 h at 39 °C, we observed a significant increase in turbidity of the cultures; then, cells were harvested by centrifugation and DNA was extracted from cell pellets. This DNA was used to construct functional metagenomic libraries in pCCFOS 1. The resulting titres of the libraries were 3.0 × 10^7^ CFUs ml^−1^ for the PSCS library; 2.3 × 10^7^ CFUs ml^−1^ for the PCS library; and 3.3 × 10^7^ CFUs ml^−1^ for the OL library. Digestions of a number of randomly chosen fosmids with *Bam*HI revealed that the restriction patterns of these clones were different, confirming that these libraries contained reasonably good DNA diversity (not shown). On average, each fosmid had an insert of about 42 kb. To ensure identification of different hemicellulases, more than 50 000 colonies per library were assayed. We found 22 clones with endoxylanase activity and 125 with β‐xylosidase activity (Table 1); this indicated that the rate of recovery of hemicellulases was in the range of 1 in 0.5 Mb, much higher than in other reports in which hemicellulases hits were in the range of 1 in 8 Mb or more (Gao *et al*., 2011; Nguyen *et al*., 2012). We consider that our high rate of success was due to the specific enrichment of rumen microbes that used industrial substrates rich in hemicellulose as a carbon and energy source. It should also be noted that the number of hemicellulase positive clones was higher in OL and PSCS libraries than in the PCS library (Table 1)— a finding that is expected given the higher hemicellulose content of OL and PSCS compared to PCS. Therefore, the combination of both *in vitro* enrichment of rumen microorganisms with industrial substrates and functional assays to identify enzymes with potential for use in biotechnological applications enhances the chances of success.

**Table 1:** Number of clones with different hemicellulolytic activities isolated from three metagenomic libraries prepared with DNA extracted from ruminal microbes after enrichment with the indicated substrate. The identification of clones with the appropriate activity in large screening assays is described in Appendix S1

Substrate used for enrichment of lignocellulosic microbes	Endoxylanase	β‐xylosidase
OL	12	66
PSCS	6	41
PCS	4	18

Fosmid DNA from the 147 clones was sequenced *en masse,* and the resulting sequences were subjected to BLAST analysis to identify hemicellulases. This approach provided very few known hemicellulases. To ensure that we were not overlooking novel hemicellulases, we BLAST searched for glycosyl hydrolase GH domains. The search revealed the presence of domains corresponding to (GH) from families 1, 5, 8, 11, 14 and 43, confirming the presence of the expected catalytic activities (Zhou *et al*., 2012; Zimbardi *et al*., 2013), although the overall identity of these sequences at the protein level was on average below 48%. BLAST analysis of the identified domains revealed certain homology with hemicellulases of microorganisms of the rumen or from anaerobic environments that is *Bryantella, Lachnospiraceae, Butyrivibrio, Fibrobacter, Prevotella, Saccharophages* and uncultured microbes (Hulo *et al*., 2006).

The positive clones were grown individually in multiwell plates, and the culture supernatants were analysed for either xylanase or β‐xylosidase activity. In the assay for endoxylanase, we used Azo‐wheat‐arabinoxylan as a substrate while in the assay for β‐xylosidase, we use *p*‐nitrophenyl‐β‐D‐xylopyranoside as a substrate; the assays were run under the conditions described in the experimental procedures with pH varying half a unit between 4.0 and 7.0 and temperatures varying 5°C between 30 and 70°C. We retained clones that were active in the pH range of 4–5.5 and temperatures over 50°C because we were searching for enzymes that could supplement cellulase‐rich fungal enzymatic cocktails that work optimally in these ranges of pH and temperatures. With these restrictions, six endoxylanase and six β‐xylosidase clones were kept for further assays.

The 12 positive clones were grown in 20 mL LB medium with chloramphenicol until they reached the stationary phase, and supernatants were then used for quantitative assays. The assays were run with the above substrates and at different pH and temperatures as above. We found that clones C5 and C20 had a xylanase activity that was at least twofold higher than that of the other four. Of these, clone C5 was superior to C20 at all pHs tested and optimal temperature was about 50 °C, being notorious that > 95% of the maximal activity was recorded in the range between 5 and 7 (Table 2). Optimal temperature was found at 50 °C, but 70% of the activity was recorded at 60 °C and near 25% activity at 70 °C. For this reason, we chose clone C5 as a source of endoxylanase for further assays (see below).

**Table 2:** Evaluation of the xylanase activity of selected metagenomic clones at different temperatures and pH. The enzyme activity was measured at different temperatures (A) for 10 min in 200 mM of sodium acetate buffer (pH 5.0). To evaluate pH (B), the assays were run at 50°C and the reaction pH values were adjusted with a citrate/phosphate buffer. Values are the average, and standard deviations of three independent replicates run in duplicate

Xylanase activity (U g^−1^protein)				
	32°C	50°C	60°C	70°C
(A)				
Cosmid 5	2740 ± 35	7080 ± 120	5270 ± 100	1900 ± 70
Cosmid 20	780 ± 220	3644 ± 100	1470 ± 170	655 ± 250

	pH 4.0	pH 5.0	pH6.0	pH 7.0
(B)				
Cosmid 5	425 ± 125	7080 ± 120	6706 ± 40	6725 ± 210
Cosmid 20	200 ± 150	3644 ± 100	1670 ± 170	1250 ± 419

Our first attempt to determine the optimal conditions for pH and temperature of the β‐xylosidase clones showed relatively low activity. The presence of β‐xylosidase in the secretome was confirmed in zymogram assays. Figure S1 depicts the zymogram in which concentrated protein is clearly visible as a single band for a few clones. As the positive clones were selected in assays run on microtitre plates, we hypothesized that oxygen levels in the test tube assay may be affecting results. To test whether this was the case, we repeated the assays under air atmosphere and under strict anaerobic conditions and found that β‐xylosidase activity was lower in the presence of oxygen. Our results revealed that clones C92 and C104 had the highest activity (Table S2 and Table 3). Fosmid DNA digestion pattern of C92 and C104 was almost identical, and we chose clone C104 for further analysis.

**Table 3:** Evaluation of β‐xylosidase activity from selected strains at different temperatures under anaerobic (A) and aerobic (B) conditions. Other conditions as in the foot note for Table 2 Values are the average, and standard deviations of three independent replicates run in duplicate

β‐xylosidase activity (U g^−1^ protein) Anaerobic (−O_2_) pH 6.0				
	32°C	50°C	60°C	70°C
(A)				
Cosmid 92	12 355 ± 200	13 610 ± 250	24 626 ± 600	830 ± 30
Cosmid 104	5960 ± 50	146 40 ± 400	28 105 ± 200	990 ± 20

β‐xylosidase activity (U g^−1^ proteins) Aerobic (+O_2_) pH 6.0				
	32°C	50°C	60°C	70°C
(B)				
Cosmid 92	4055 ± 600	6900 ± 144	8970 ± 45	3020 ± 400
Cosmid 104	3170 ± 100	7175 ± 262	7410 ± 90	2770 ± 280

First, we sequenced the selected fosmid clones (xylanase C5 and β‐xylosidase C104). Sequence analysis of fosmid C5 revealed an insert of about 45 kb in size. BLAST analysis revealed a number of ORFs that exhibited an identity range between 34 and 98% to translated sequences of a cellulolytic bacterium present in the rumen of goats known as *Fibrobacter succinogenes* (Table S3). Of these sequences, we identified a putative ORF that shared 59% similarity with an *Fibrobacter succinogenes* endo‐1,4‐β‐xylanase (Bae *et al*., 1993; Fukuma *et al*., 2015). PROSITE analysis of this translated polypeptide identified two domains, one spanning residues 45 to 254 and another spanning residue 309 to 508, which are believed to be part of the GH family 11 (GH11) active domain (Hulo *et al*., 2006). GH11 is a family of mono specific enzymes that only have xylanase activity and lack of cellulase activity. Analysis of the sequence of fosmid C104 revealed an insert of about 40 kb with a long contig of 31 kb and a number of smaller contigs. BLAST analysis identified a β‐xylosidase ORF that presented 63% identity with an enzyme from *Lacchnospiraceae bacterium* NK4A179 (Table S3).

To further confirm that the two ORFs identified above encode an endoxylanase and a β‐xylosidase, the corresponding genes were amplified by PCR using appropriate primers (See Suporting Information), cloned into pET24b and expressed in *E. coli* BL21 with an N‐terminal 6× histidine tag. The recombinant proteins were purified to homogeneity, as described in Appendix S1. Overexpressed histidine‐tagged xylanase C5 protein ran as a single protein band that migrated at 66 kDa in a Coomassie‐stained gel and exhibited endoxylanase activity, with the maximum activity levels recorded at 50°C and pH 5, confirming the results obtained with the fosmids. The overexpressed histidine‐tagged β‐xylosidase C104 protein ran as a protein band that migrated at 74.6 kDa in SDS polyacrylamide gels, and its maximum specific activity was recorded at 60°C and pH 5.5.

Our assays showed that maximal activity of the xylanase and β‐xylosidase characterized in this work was at around pH 5 and at 50°C to 60 °C, lower pH and higher temperatures than that in the niche where microbes proliferate; this is not surprising as growth of microbes and rumen activities are optimized rather than maximized. It is worth highlighting that the optimal pH and temperature at which these enzymes function favour our aim of using these enzymes in 2G ethanol production. It is also worth to mention that the above specific activities are much higher than the values we have measured using industrial fungal cocktails or those reported by others (Hayashi *et al*., 2001; Saha, 2001, 2003; Collins *et al*., 2005).

### Effect of enzyme supplementation on the enzymatic hydrolysis of PCS and PSCS biomass

To test the effect of supplementation of a fungal enzymatic cocktail with bacterial enzymes, we measured sugars released from pretreated sugarcane straw. Sugars were quantified using HPLC analysis. All enzymatic cocktails had a protein concentration of 10 mg protein per *g* of glucan in the PSCS. The cocktail was made of 9 mg protein g^−1^ glucan from a preindustrial *M. thermophila* strain supplemented with 1 mg protein per *g* glucan of a mixture of xylanase C5 and β‐xylosidase C104. Controls with no added bacterial enzymes or with just the fungal cocktail were also run. The assays were run at pH 5.5 and 50°C for 72 h. We observed an increase in the release of glucose and xylose (about 15%) from PSCS when bacterial enzymes were added (Table 4). In terms of g per kg of initial biomass, the increases were 7.8 ± 1.0 and 4.4 ± 0.5 g kg^−1^ of glucose and xylose respectively. Concomitantly, we found a decrease in the level of xylobiose corresponding to 3.3 g kg^−1^. These results support that both cellulose and hemicellulose were better broken down when the fungal cocktail was supplemented with ruminal hemicellulases, this comes as a surprise as we expected only better on digestion of hemicellulose. Based on these results, we propose that the enhanced breakdown of hemicellulose fibres favours the enzymatic attack of cellulose by fungal cellulases, demonstrating the synergism of enzymes from eukaryotes and prokaryotes in lignocellulose conversion into monosaccharides. In natural habitats, plant biomass cell walls are degraded by a variety of lignocellulosic microbes including fungi and bacteria that breakdown them synergistically (Shallom and Shoham, 2003; Hori *et al*., 2014), therefore, our results with enzymatic cocktails showed that we can reproduce in test tubes the observations made in nature.

**Table 4:** Enzymatic hydrolysis of PSCS substrate with supplementation of β‐xylanase/β‐xylosidase. It is shown the amount of sugar released in g per kg dry. Purified proteins were added at 1 mg g⁻¹ glucan to an enzymatic mixture of the preindustrial *M. thermophila* strain at 9 mg g^−^¹ glucan. The mixture was incubated at 50°C and pH 6 for 72 h with shaking (150 rpm)

	Glucose	Xylose	Xylobiose
Fungal	48.1 ± 4	26.2 ± 6	16.1 ± 6
Fungal+Bacterial	55.9 ± 5	30.6 ± 0	12.8 ± 2

## Conflict of interest

None declared.

## Supporting information


**Appendix S1.** We sought new hemicellulases using ruminal liquid, after enrichment of microbes with industrial lignocellulosic substrates and preparation of metagenomic libraries. Among 150 000 fosmid clones tested, we identified 22 clones with endoxylanase activity and 125 with b‐xylosidase activity. These positive clones were sequenced en masse and the analysis revealed open reading frames with a low degree of similarity with known glycosyl hydrolases families.
**Fig S1.** Electrophoretic separation of protein secreted by metagenomic library clones.
**Table S1.** Compositional analysis of industrial substrates.
**Table S2.** Specific endoxylanase and β‐xylosidase activity in assays run at pH 5.0 and 50°C with supernatants from the indicated clones.
**Table S3.** Annotation of genes in cosmid C5 insert retrieved from a metagenomic library prepared from goat's rumen metagenomic libraries.
**Table S4.** Annotation of genes in cosmid C104 insert retieved from a metagenomic library prepared from goat's rumen metagenomic libraries.
