# Supplementary material for: Ruminal metagenomic libraries as a source of relevant hemicellulolytic enzymes for biofuel production
Source: Microb Biotechnol. 2018 Apr 17;11(4):781–7. doi: 10.1111/1751-7915.13269 (PMC6011990; doi:10.1111/1751-7915.13269)
Supplement: Supplementary file 1 — Appendix S1. We sought new hemicellulases using ruminal liquid, after enrichment of microbes with industrial lignocellulosic substrates and preparation of metagenomic libraries. Among 150 000 fosmid clones tested, we identified 22 clones with endoxylanase activity and 125 with b‐xylosidase activity. These positive clones were sequenced en masse and the analysis revealed open reading frames with a low degree of similarity with known glycosyl hydrolases families. Fig S1. Electrophoretic separation of protein secreted by metagenomic library clones. Table S1. Compositional analysis of industrial substrates. Table S2. Specific endoxylanase and β‐xylosidase activity in assays run at pH 5.0 and 50°C with supernatants from the indicated clones. Table S3. Annotation of genes in cosmid C5 insert retrieved from a metagenomic library prepared from goat's rumen metagenomic libraries. Table S4. Annotation of genes in cosmid C104 insert retieved from a metagenomic library prepared from goat's rumen metagenomic libraries. [file MBT2-11-781-s001.doc]

**SUPPLEMENTARY INFORMATION**

*Animals and substrates*

Three rumen-cannulated Murciano-Granadina goats (46.5 ± 2.9 kg body weight) were used as a source of ruminal contents for the enrichment of microbes using *in vitro* incubations. Animals were cared for and handled by trained personnel in accordance with the Spanish guidelines for experimental animal protection (Royal Decree 53/2013 of February 1st on the protection of animals used for experimentation or other scientific purposes). All the experimental procedures were approved by the Animal Welfare Committee at the Estación Experimental del Zaidin (CSIC, Spain). Animals were fed a standard diet composed of alfalfa hay and a commercial concentrate in a 1:1 ratio (Prieto et al., 1990). Clean water and mineral supplements were always available.

*Experimental procedures and sampling:*

*In vitro* incubations

The lignocellulosic materials used in this study were pretreated corn stover (PCS), pretreated sugar cane straw (PSCS) and olive branches and leaves (OL). The chemical composition of the substrates is shown in Suppl. Table 1. Substrates were milled and dried at 56°C for 48 h.

The rumen content from each of the 3 goats was collected into thermal flasks before the morning feeding, pooled and immediately taken to the laboratory. The rumen contents were strained through four layers of cheesecloth and mixed with a buffer solution (Goering and Van Soest, 1970) in a 1:4 v/v ratio at 39°C under continuous flushing with CO_2_. The time required from rumen content collection to inoculation of bottles was < 30 min. Fifty milliliters of the buffered rumen fluid were anaerobically added into 120 mL bottles. The bottles were sealed with butyl rubber stoppers and aluminum caps and incubated at 39ºC in a water bath. Three 72h incubation runs were carried out using bottles of 120 mL of volume. In each run 3 bottles were incubated per substrate (500 mg/substrate/bottle). Additionally, 3 bottles with a standard substrate and 3 as blanks (without substrate) were incubated. Gas produced during fermentation was extracted from the bottles 4 times a day using a glass-calibrated syringe (Ruthe®, Normax, Marinha Grande, Portugal). After this 72h of incubation, fermentation was stopped by placing the bottles in ice and half of the content of each bottle was then stored at -20ºC and the other half at -80ºC.

*DNA extraction and construction of fosmid libraries*

DNA was extracted from ruminal samples following *in vitro* incubation for 72 h at 39ºC in anaerobic conditions. Microbial cells were removed from the substrates by shaking the samples with glass beads. Samples were then filtered and centrifuged. DNA was isolated using the Genome DNA kit (Genomic DNA Kit, MP Biomedicals) following the manufacturer´s instructions. High molecular weight DNA was isolated from gels as 40 kb fragments DNA was then end-repaired and ligated into the blunt-end cut pCCFOS1 vector (Epicenter). The resulting product was transduced into *E. coli* EPI300-T1R and plated onto LB-agar plates supplemented with chloramphenicol (Cm). The presence of inserts of the appropriate size in the fosmids was verified with the digestion of 20 randomly chosen individual clones. Libraries were kept as glycerol stocks at -80 ºC.

*Screening for hemicellulolytic activities*

Metagenomic libraries were screened on solid medium (245 x 245 mm plates) containing LB-agar supplemented with 30 mg l^−1^ Cm, 0.01% (w/v) arabinose (to induce fosmid replication) and, 0.05% (w/v) Azanin-Cross-linked-xylan (AZCL-xylan, Sigma-Aldrich) or 0.2 g l^-1^ of *p*-Nitrophenyl-β-*D*-xylopyranoside (pNXP, Megazyme) for the identification of clones expressing endoxylanase and β-xylosidase activity, respectively. At least 150,000 clones were assayed per substrate. Plates were incubated at 37°C for 24 to 48 h. Endoxylanase activity was visualized as a blue color around a colony, whereas β-xylosidase was visualized as a yellow halo. The positive colonies obtained were restreaked on the same composition agar plate to confirm the original phenotype.

*Enzymatic assays*

*Escherichia coli* EPI300*-*T1R bearing positive fosmid clones were grown in LB liquid medium with shaking at 37ºC, with 30 mg/mL chloramphenicol (Cm) and arabinose 0.01% (w/v). Cultures were harvested by centrifugation and supernatants were used as a source of endoxylanase and β-xylosidase activities. The protein concentration was determined by using the Compat-Able Protein Assay Preparation Reagent Set (Thermo Scientific) coupled with the bicinchoninic acid assay (Smith *et al*., 1985).

The standard β-Xylosidase (BXL) activity assay was in 200 mM acetate buffer (pH 5.0) and 50ºC with 0.2 g l^-1^ *p*-Nitrophenyl-β-*D*-xylopyranoside (pNXP, Sigma) as a substrate . Absorbance was measured at 410 nm after 10 minutes of incubation. Activity was calculated based on the molar extinction coefficient of *p*-nitrophenol.

The standard assay for Endoxylanase (XYL) activity was carried out at 50°C for 10 minutes in 200 mM sodium acetate buffer pH 5.0 with 1% (w/v) Azo-wheat-arabinoxylan (Azo-Wax, Megazyme). After incubation, ethanol (95%) was added to stop the reaction and the supernatant was collected by centrifugation. Absorbance was measured at 590 nm and the activity was calculated by reference to a standard curve produced with an endo-xylanase of *Aspergillus niger*, according to the supplier of the substrate. One unit of enzyme activity is defined as the amount of enzyme required to release one micromole of xylose reducing-sugar equivalents from wheat arabinoxylan.

*Sequence analysis of selected fosmids*

Positive fosmid clones with a range of cellulolytic and hemicellulolytic activities were pooled and sequenced. In addition, the fosmid clones that yielded the highest xylanase (C5) and β-xylosidase (C104) activities were sequenced by StabVida, NGS Laboratory (Lisbon, Portugal). Sequencing reactions of 600 cycles were performed on a Personal Genome Machine using the Ion Hi-Q Sequencing kit and one 314 chip. The raw data were analyzed using the CLC Genomics Workbench 8.0.1 program. Final assembled contigs from fosmid reads were analyzed on a Centos6 computer using BlastX (Altschu*l et* al., 1997) versus the NCBI’s protein database (hppt://www.ncbi.nlm.gov/BLAST/) or a local BlastX search using a non-redundant Uniprot protein database.

##### Cloning and purification of selected recombinant proteins in E. coli

Endo-xylanase C5 and β-xylosidase C104 genes were PCR amplified using primers designed from target sequences:

*NdeI*Cos5.Xylanasa1.F: 5′-CATATGAAAAACATTTCTAGTGTAGCCAAGATAGGCTT-3′ (forward)

*XhoI*Cos5.Xylanasa1.R: 5′-CTCGAGTTACTTCACGGAGAAGCGCTGC-3′ (reverse)

Cos104*Nhe*I: 5´- GCTAGCGGAATGCCGGTGAAATATTATGTAGATTGTAAAG-3´ (forward)

Cos104*Not*I: 5´-GCGGCCGCTGCAAACGGGCCCGCGATCACTTCACCTTCAC-3´ (reverse)

Upon amplification, the fragments were cloned into the pMBL vector to yield pMBL::xyl-5 and pMBL::bxyl-104, respectively. The plasmid pMBL::xyl-5 was subsequently digested with *Nde*I*/Xho*I and the plasmid pMBL::bxyl-104 with *Nhe*I/*Not*I, and then the corresponding fragments cloned into pET24b(+) previously digested with the same enzymes. The resulting plasmids, pET24b::xyl-5 and pET24b::bxyl-104 were transformed into *E. coli* BL21 (DE3) and used to express the endoxylanase and β-xylosidase proteins, respectively, with a polyhistidine tag at their N-termini. Cells were grown in 2 liter conical flasks with 500 ml of LB supplemented with 50 μg ml^-1^ kanamycin. Cultures were incubated at 37°C and shaken until they reached a turbidity of 0.6 at 660 nm. Then IPTG (0.5-1 mM) was added to induce the expression of the cloned genes and incubation continued at 30°C. After overnight incubation, the cells were harvested by centrifugation (30 min at 20,000 × g) and the supernatant containing the protein was supplemented with glycerol at 20% (v/v) and stored for protein purification.

For protein purification, supernatants were concentrated with Vivaspin 20 (Sartorius stedim), filtered (0.45 µm pore diameter) and the filtrate loaded onto a 5-ml His-Trap chelating column (GE Healthcare) previously equilibrated with buffer A (50 mM Tris-HCl, pH 7.9, 300 mM NaCl, 1 mM dithiothreitol, 10 mM imidazole) supplemented with a tablet of Complete^TM^ EDTA-free protease inhibitor mixture (Roche Applied Science). The proteins were eluted using a 1 to 500 mM gradient of imidazole in buffer A. The purity of the eluted proteins was analyzed using 12% (w/v) SDS-PAGE gels.

*Native electrophoresis gels and zymogram assays of β-xylosidase.*

For zymogram studies of fosmid-encoded β-xylosidase secreted proteins, protein was concentrated using a centrifugal concentrator (Vivaspin 20, Sartorius stedim). About 50 µg of non-heated protein was loaded in parallel in two 7.5 % (w/v) polyacrylamide gels, separated at 100 mV, as described by [Brunelle and Green (2014)](#_ENREF_7). The gels were then washed in 10 ml of Triton 2.5% (v/v) for 30 min and incubated in 0.1 M sodium acetate buffer (pH 5) for 5 minutes; one of the gels was stained with Coomassie blue to visualize proteins, while the other was incubated with 0.1 ml of MU-Xylopyranoside (4-Methylumbelliferyl-β-D-xylopyranoside, Sigma-Aldrich) at 1 mg ml^-1^ for 5 minutes.

### *Enzymatic hydrolysis of biomass (PSCS or PCS)*

PCS and PSCS were used as substrates and the pH of the suspension was adjusted to 5.5 by supplementation of ammonium hydroxide. The fungal enzymatic cocktail used was prepared from a pre-industrial *Myceliophthora thermophila* strain that secreted PMOs, endoglucanases, exoglucanases and β-glucosidase and it was added at 9 mg per g of glucan to PCS or PSCS. When xylanase or β-xylosidase was added they were supplied at a concentration of 1 mg per *g* of glucan. The different enzymatic cocktails were incubated at pH 5.5 and 50°C for 72 h with shaking (150 rpm). Hydrolysis was also carried out under either under air or under a nitrogen atmosphere, in the latter case all of the samples were prepared in an anaerobic cabinet. After enzymatic hydrolysis, samples were filtered and analyzed by high-performance liquid chromatography (HPLC) using an Aminex HPX-87H (300×7.8 mm) column with 9 μm particle size (Bio-Rad). The analyses were performed at 60°C under isocratic conditions with 5 mM H_2_SO_4_ as the mobile phase at a flow rate of 0.6 ml min^-1^ with an injection volume of 20 μl. Carbohydrates were analyzed using a refractive index detector (Bermúdez-Alcántara *et al.,* 2016). The total reducing sugars released were used to calculate hydrolysis yields.

**REFERENCES**

Altschul, S., Madden, T., Schaffer, A., Zhang, J., Zhang, Z., Miller, W. and Dj, L. (1997) Gapped BLAST and PSI- BLAST: a new generation of protein database search programs. *Nucleic Acids Res*, **25**: 3389-3402.

Bermudez-Alcántara, M.A., Dobruchowska, J., Azadi, P., Díez, B., Molina‑Heredia, F. and Reyes‑Sosa, F.M. (2016) Recalcitrant carbohydrates after enzymatic hydrolysis of pretreated lignocellulosic biomass. *Biotech Biofuels*, 9:207.

Brunelle, J.L. and Green, R. (2014) One-dimensional SDS-polyacrylamide gel electrophoresis (1D SDS-PAGE). *Methods Enzymol*, **541**: 151-159.

Goering, H.K., Van Soest, P.J., (1970). Forage fiber analysis (apparatus, reagents, procedures and some applications). *Agricultural Handbook* no. 379. US Department of Agriculture, Washington.

Prieto, C., Aguilera, J.F., Lara, L., Fonollá, J. (1990). Protein and energy requirements for maintenance of indigenous Granadina goats. *British J Nut*, **63**: 155–163.

Smith, P.k., Krohn, R.I., Hermanson, G.T., Mallia, A.K., Gartner, M.D., Provenzano, E.K., *et al*., (1985) Measurement of protein with bicinchoninic acid. *Anal Biochem* **150:** 76-85.

Zimbardi, A.L., Sehn, C., Meleiro, L.P., Souza, F.H., Masui, D.C., Nozawa, M.S., et al. (2013) Optimization of beta-glucosidase, β-xylosidase and xylanase production by *Colletotrichum graminicola* under solid-state fermentation and application in raw sugarcane trash saccharification. *Int J Mol Sci* **14**: 2875-2902.

**Supplementary Tables**

| **Substrate** | **Dry material g/100 g fresh material** | **Cellulose** | **Hemi**  **cellulose** | **Neutral detergent fiber** | **Acid detergent fiber** | **Acid detergent lignin** | **Soluble in neutral detergent** |
| --- | --- | --- | --- | --- | --- | --- | --- |
| **PCS** | 37.8 | 37.3 | 3.5 | 51.5 | 48.0 | 10.7 | 48.5 |
| **PSCS** | 32.9 | 16.1 | 18.5 | 39.9 | 21.4 | 5.3 | 60.1 |
| **OL** | 72.8 | 31.9 | 13.7 | 48.5 | 34.8 | 2.9 | 51.5 |

**Table S1.** Compositional analysis of industrial substrates

| **Clone number** | **Endoxylanase**  **(U/mgr protein)** | **Β-xylosidases**  **(U/mg protein)** |
| --- | --- | --- |
| **C5** | **3.500** | **_** |
| **C7** | **1.800** | **_** |
| **C19** | **_** | **900** |
| **C20** | **3.500** | **-** |
| **C35** | **500** | **_** |
| **C67** | **700** | **_** |
| **C92** | **_** | **6.000** |
| **C104** | **_** | **7.000** |
| **C110** | **_** | **300** |
| **C120** | **1.700** | **800** |
| **C131** | **_** | **1.300** |

**Table S2.** Specific endoxylanase and β-xylosidase activity in assays run at pH 5.0 and 50º C with supernatants from the indicated clones.

| **Cosmid 5** | | | | |
| --- | --- | --- | --- | --- |
| **Gene id** | **e-value** | **Identity %** | **Protein name** | **Organism** |
| FSU_0750 | 2E-043 | 60 | DNA-binding protein | *Fibrobacter succinogenes* |
| FSU_0751 | 0,0 | 97 | 23s rRNA | *Fibrobacter succinogenes* |
| FSU_0752 | 9E-139 | 99 | Putative thiol methyltransferase | *Fibrobacter succinogenes* |
| FSU_0753 | 6E-099 | 97 | SEC-C domain protein | *Fibrobacter succinogenes* |
| FSU_0754 | 0,0 | 95 | N-acetyl-gamma-glutamyl-phosphate reductase | *Fibrobacter succinogenes* |
| FSU_0755 | 2E-010 | 34 | Putative lipoprotein | *Fibrobacter succinogenes* |
| FSU_0758 | 1E-016 | 47 | TonB family protein | *Fibrobacter succinogenes* |
| FSU_0759 | 1E-026 | 45 | Conserved domain protein | *Fibrobacter succinogenes* |
| FSU_0761 | 0,0 | 94 | GTP-binding protein Obg/CgtA | *Fibrobacter succinogenes* |
| FSU_0762 | 2E-065 | 91 | DNA binding protein HU | *Fibrobacter succinogenes* |
| FSU_0763 | 1E-102 | 96 | SsrA binding protein | *Fibrobacter succinogenes* |
| FSU_0764 | 0,0 | 85 | N-acetyl muramoyl L-alanine amidase family | *Fibrobacter succinogenes* |
| FSU_0765 | 5E-050 | 40 | Putative lipoprotein | *Fibrobacter succinogenes* |
| FSU_0767 | 3E-117 | 80 | Putative lipoprotein | *Fibrobacter succinogenes* |
| FSU_0771 | 1E-151 | 99 | DNA-binding regulatory protein | *Fibrobacter succinogenes* |
| FSU_0772 | 1E-118 | 87 | Hypothetical protein | *Fibrobacter succinogenes* |
| FSU_0773 | 0,0 | 99 | Phosphopglucomutase | *Fibrobacter succinogenes* |
| FSU_0774 | 2E-126 | 84 | Conserved domian protein | *Fibrobacter succinogenes* |
| FSU_0775 | 0,0 | 99 | Dihydrodipicolinate synthase | *Fibrobacter succinogenes* |
| FSU_0777 | 0,0 | 59 | Endo 1,4 beta-xylanase | *Fibrobacter succinogenes* |
| FSU_0778 | 0,0 | 97 | gyrA | *Fibrobacter succinogenes* |
| FSU_0779 | 1E-171 | 94 | Acid phgosphatase surE | *Fibrobacter succinogenes* |
| FSU_0780 | 2E-160 | 98 | RNA methyltransferase | *Fibrobacter succinogenes* |

**Table S3.** Annotation of genes in cosmid C5 insert retrieved from a metagenomic library prepared from goat’s rumen metagenomic libraries.

| **Cosmid 104** | | |
| --- | --- | --- |
| **Domain** | **E-value** | **Protein name** |
| COG2316 | 5.04e-18 | Predicted hydrolase, HD superfamily |
| YdcF-like | 6.39e-11 | YdcF-like. YdcF-like is a large family of mainly bacterial proteins |
| GH43_AXH_like | 3.84e-21 | Glycosyl hydrolase family 43 |
| MtbC1 | 2.72e-44 | Methanogenic corrinoid protein MtbC1 [Energy production and conversion] |
| trimeth_pyl | 0 | trimethylamine:corrinoid methyltransferase |
| Cob-chelat-sub | 4.05e-103 | cobaltochelatase subunit |
| Cob-chelat-sub | 4.03e-27 | cobaltochelatase subunit |
| ChlD | 8.82e-39 | Mg-chelatase subunit ChlD [Coenzyme transport and metabolism] |
| PotE | 4.79e-16 | Amino acid transporter [Amino acid transport and metabolism] |
| HMG_CoA_R_NADP | 1.31e-103 | 3-hydroxy-3-methylglutaryl Coenzyme A reductase |
| Cation_efflux | 2.08e-24 | Cation efflux family |
| pheT | 3.98e-161 | phenylalanyl-tRNA synthetase subunit beta |
| Bcp | 5.85e-36 | Peroxiredoxin [Posttranslational modification, protein turnover, chaperones] |
| SHMT | 2.34e-72 | Serine-glycine hydroxymethyltransferase (SHMT) |
| ribB | 9.54e-37 | 3,4-dihydroxy-2-butanone 4-phosphate synthase |
| TagD | 3.96e-30 | Glycerol-3-phosphate cytidylyltransferase. |
| PRK06455 | 1.09e-68 | riboflavin synthase; Provisional |
| Lumazine synthase | 4.53e-45 | lumazine synthase (6,7-dimethyl-8-ribityllumazine synthase, LS) |
| MntP | 5.35e-37 | Putative Mn2+ efflux pump MntP [Inorganic ion transport and metabolism] |
| NorV | 5.96e-61 | Flavorubredoxin [Energy production and conversion] |
| NapF | 1.02e-04 | Ferredoxin [Energy production and conversion] |
| INT_tnpA_C_Tn554 | 3.81e-03 | Putative Transposase A from transposon Tn554, C-terminal catalytic domain |
| Rotamase_3 | 6.81e-22 | PPIC-type PPIASE domain |

**Table S4.** Annotation of genes in cosmid C104 insert retieved from a metagenomic library prepared from goat’s rumen metagenomic libraries.

**
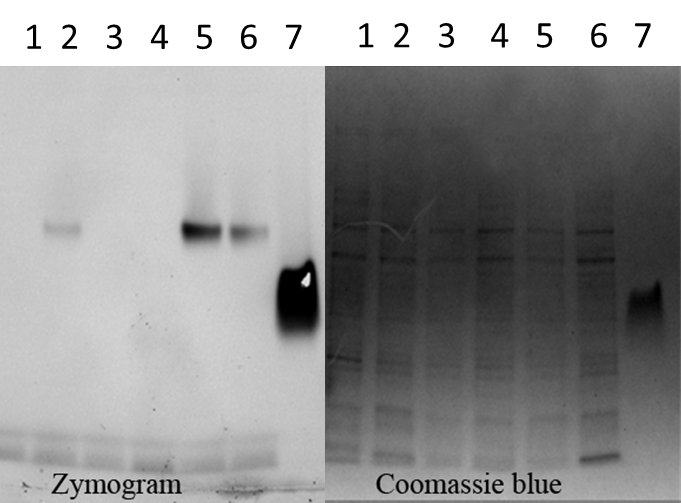
**

**Supplementary Figure 1.** Electrophoretic separation of protein secreted by metagenomic library clones. Samples were run as decribed in Supplementary information in 7.5% SDS-PAGE gels at 120 mV for 1h. Right panel is a coomassie blue stain gel; while the left panel is a zymogram assay. Lanes 1 through 6 correspond to selected clones with β-xylosidase activity. Lane 7 corresponds to purified β-xylosidase protein from *Fusarium sp*.
